# Bioaccessibility of Tocols in Commercial Maize Hybrids Determined by an In Vitro Digestion Model for Poultry

**DOI:** 10.3390/molecules28135015

**Published:** 2023-06-27

**Authors:** Veronika Gunjević, Dora Zurak, Darko Grbeša, Goran Kiš, Tatjana Međimurec, Vasil Pirgozliev, Kristina Kljak

**Affiliations:** 1 Faculty of Agriculture, University of Zagreb, Svetošimunska Cesta 25, 10000 Zagreb, Croatia; vgunjevic@agr.hr (V.G.); dzurak@agr.hr (D.Z.); dgrbesa@agr.hr (D.G.); kis@agr.hr (G.K.); 2 Ministry of Agriculture, Directorate for Professional Support to the Development of Agriculture and Fisheries, Bani 110, Buzin, 10010 Zagreb, Croatia; tatjana.medimurec@mps.hr; 3 National Institute of Poultry Husbandry, Harper Adams University, Newport TF10 8NB, UK; vpirgozliev@harper-adams.ac.uk

**Keywords:** maize, commercial hybrids, tocols, bioaccessibility, in vitro digestion

## Abstract

Despite the high proportion of maize grain in animal diets, the contribution made by maize phytochemicals is neglected. Tocols and their contribution to the vitamin E content of animal diets are one example, exacerbated by sparse information on the tocol bioaccessibility of commercial hybrids. In this study, the contents of individual and total tocols and their bioaccessibility were determined in the grain samples of 103 commercial hybrids using a standardized INFOGEST digestion procedure. In the studied hybrids, total tocol content ranged from 19.24 to 54.44 µg/g of dry matter. The contents of micellar α-, γ-, δ-tocopherols, γ-tocotrienol, and total tocols correlated positively with the corresponding contents in the grain samples of the studied hybrids. In contrast, a negative correlation was observed between the bioaccessibility of γ- tocopherol, α- and γ-tocotrienol, and total tocols, along with the corresponding contents in the grain of studied hybrids. The highest bioaccessibility was exhibited by γ-tocotrienol (532.77 g/kg), followed by δ-tocopherol (529.88 g/kg), γ-tocopherol (461.76 g/kg), α-tocopherol (406.49 g/kg), and α-tocotrienol (359.07 g/kg). Overall, there are significant differences in the content and bioaccessibility of total and individual tocols among commercial maize hybrids, allowing the selection of hybrids for animal production based not only on crude chemical composition but also on the content of phytochemicals.

## 1. Introduction

The term vitamin E refers to a group of naturally occurring compounds (tocols) that can occur in 8 different forms, specifically, α-, β-, γ-, and δ-tocopherols and α-, β-, γ-, and δ-tocotrienols [1,2], all of which act as antioxidants in membranes and plasma lipoproteins. Within the tocols, α-tocopherol is primarily found in green leafy plants and conserved forage, while γ-tocopherol is the main tocol in many seeds and their products [3]. Due to their strong antioxidant properties, tocols can prevent oxidative damage to cells [4]. Vitamin E also has an anti-inflammatory effect and influences the expression of the so-called vitamin E-related genes [5]. These biological activities may prevent cancer, cardiovascular diseases, neurological disorders, inflammatory diseases, and several age-related degenerative diseases [4,6]. In animal plasma, α-tocopherol is the predominant and most active form of vitamin E [7], reversing the symptoms of hypovitaminosis E [8].

Humans and animals cannot synthesize vitamin E; therefore, they rely on dietary sources for these compounds [9]. In addition to being used for their biological functions, tocols can be deposited in animal tissues such as muscles, fats, and egg yolks. Recently, eggs have been studied as a functional food and were found to be an ideal vehicle for the biofortification of vitamins, folic acid, selenium, polyunsaturated fatty acids, ω-3 fatty acids, and carotenoids [10,11,12]. Nowadays, consumers are concerned about the use of synthetic additives and there is a growing tendency to replace them with natural alternatives. Maize could be one such alternative for use as an ingredient in complete feeds for poultry.

Maize is the most widely used cereal in complete feeds for poultry [13,14] and is recognized as one of the most important cereals [4]. It contains numerous micronutrients, among which tocopherols and tocotrienols are relatively abundant maize antioxidants [15]. Large differences in tocol content have been demonstrated in different genotypes [4,16]: however, information on their potential biological utilization is scarce.

Both the bioaccessibility and bioavailability of a bioactive compound that is incorporated in food/feed are of great importance since they allow a phytochemical to exert its bioactivity after ingestion. The digestion efficiency of highly lipophilic food micronutrients such as tocols depends on numerous factors and varies greatly between food matrices [17]. After ingestion, tocols are released from the food and are then incorporated into micelles, which makes them bioaccessible. The micelles transport them to the epithelial cells, where their absorption occurs [6]. Bioaccessibility is defined as the ratio of the compound found in the mixed micelles formed during digestion, relative to the initial content of the compound in the matrix [18]. Thus, the bioaccessibility of a compound implies more about its bioactivity than its content in the matrix alone.

Due to the scarcity of studies reporting tocol content in commercial maize hybrids and tocol bioaccessibility from maize in general, the main objective of this study was to evaluate the bioaccessibility of individual and total tocols in 103 maize hybrids that were available on the Croatian market, sowing these hybrids at the test field. The bioaccessibility of tocols was evaluated in an in vitro model using a standardized INFOGEST digestion procedure. Due to the aforementioned significance of eggs as functional foods, the in vitro model used to study the bioaccessibility of maize tocols was adapted to mimic their ingestion by poultry.

## 2. Results

### 2.1. Tocol Content in Studied Maize Hybrids

Among all the studied maize hybrids, total tocol content ranged from 19.24 to 54.44 µg/g of dry matter (DM), demonstrating a wide variability in tocol content among hybrids and allowing their classification into six hybrid groups (G1–G6). The percentage of hybrids present in each specific group is shown in Table 1. The contents of individual and total tocols for each maize hybrid that was studied are given in the Supplementary Materials of this paper.

**Figure 1:**
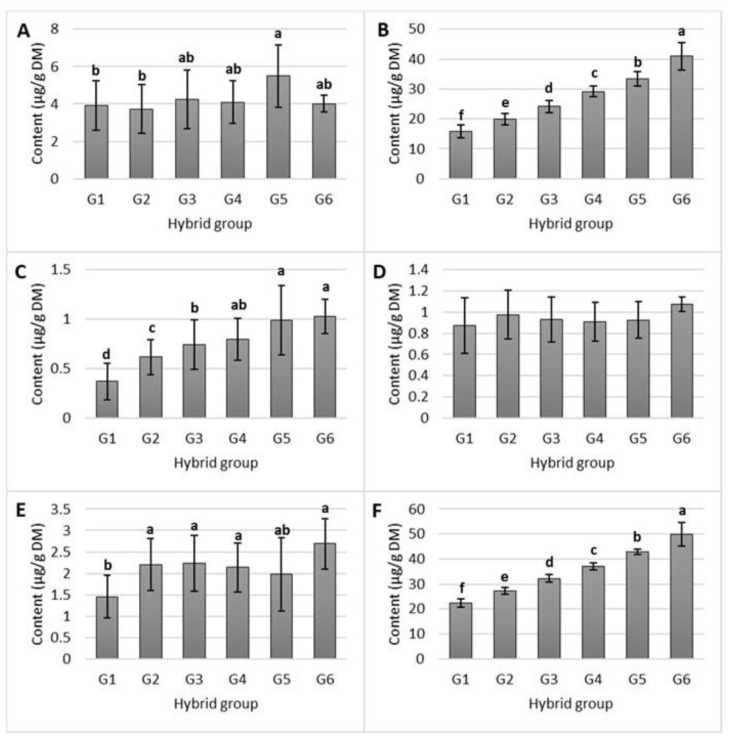
Contents of α-tocopherol (**A**), γ-tocopherol (**B**), δ-tocopherol (**C**), α-tocotrienol (**D**), γ-tocotrienol (**E**), and total tocols (**F**) in the different maize hybrids, which have been categorized into six groups (G) based on their total tocol content (G1 < 25, G2 25–30, G3 30–35, G4 35–40, G5 40–45, and G6 > 45 μg/g DM). Results are expressed as average values ± SD. Statistically different data (*p* < 0.05) are indicated by lower-case letters (a–f).

The predominant vitamin E derivative in all the studied maize hybrids was γ-tocopherol; its average proportion was 75.28% of the total tocols. γ-tocopherol was followed by α-tocopherol (12.84% of total tocols), γ-tocotrienol (6.72% of total tocols), α-tocotrienol (2.97% of total tocols), and δ-tocopherol (2.19% of total tocols). The content ranges (µg/g DM) were from 1.77 to 8.66 for α-tocopherol, from 12.10 to 45.24 for γ-tocopherol, from 0.16 to 1.74 for δ-tocopherol, from 0.25 to 1.40 for α-tocotrienol, and from 0.82 to 3.68 for ƴ-tocotrienol. The contents of the individual tocols between hybrid groups varied significantly for all tocols except α-tocotrienol (*p* < 0.05; Figure 1). Furthermore, the contents of γ-tocopherol, δ-tocopherol (*p* < 0.0001), and γ-tocotrienol (*p* < 0.05) in the hybrid groups increased linearly with the increasing total tocol content. The remaining tocols showed no linear relationship with the total tocol content.

### 2.2. Bioaccessibility of Tocols in the Studied Maize Hybrids

The contents of digestible γ-tocopherol, δ-tocopherol, and total tocols increased linearly with the increase in the hybrid group of tested samples (*p* < 0.0001). Therefore, a positive correlation was found between the contents of γ-tocopherol, δ-tocopherol, and total tocols in the grain and in the digesta after performing an in vitro digestion procedure (Table 2). A positive correlation between the contents in the grain and the digesta was also found for δ-tocopherol and γ-tocotrienol.

**Figure 2:**
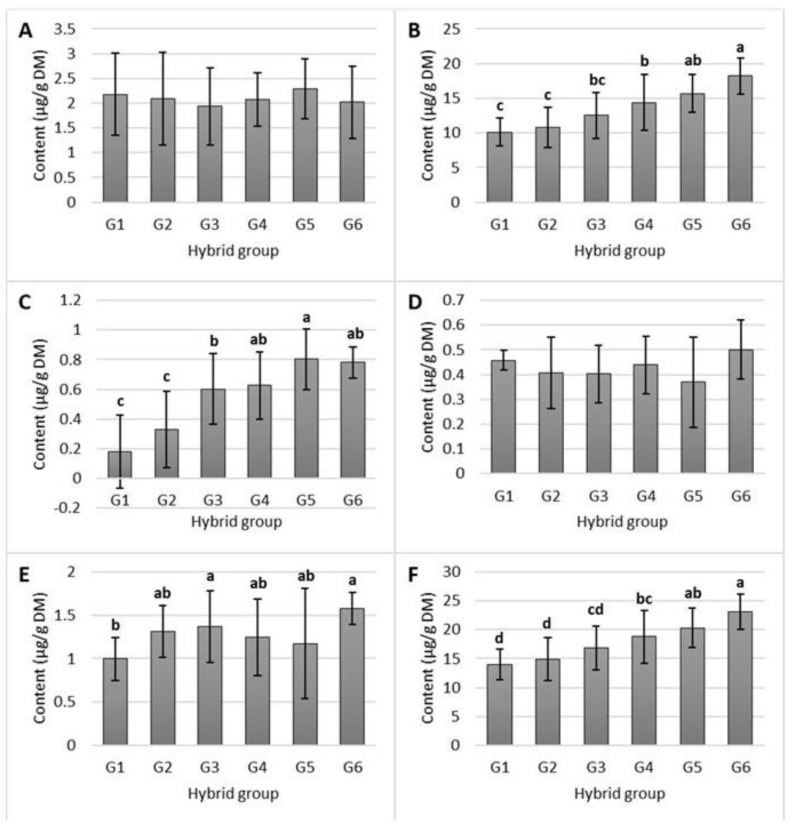
Contents of digestible α-tocopherol (**A**), γ-tocopherol (**B**), δ-tocopherol (**C**), α-tocotrienol (**D**), γ-tocotrienol (**E**), and total tocols (**F**) in the different maize hybrids, which have been categorized into six groups (G) based on their total vitamin E contents (G1 < 25, G2 25–30, G3 30–35, G4 35–40, G5 40–45, and G6 > 45 μg/g DM). The presented values that are followed by different lower-case letters (a–d) are significantly different from each other (*p* < 0.05).

The contents of tocols in the digesta (µg/g DM) ranged from 0 to 4.68 for α-tocopherol, from 6.41 to 24.26 for γ-tocopherol, from 0 to 1.08 for δ-tocopherol, from 0 to 0.71 for α-tocotrienol, and from 0.49 to 2.56 for ƴ-tocotrienol (Figure 2). The digestibility, i.e., the ratio between the contents of digestible tocols and tocols in maize grain, decreased in the following order: δ-tocopherol (682.52 g/kg) > γ-tocotrienol (620.04 g/kg) > γ-tocopherol (524.09 g/kg) > α-tocopherol (523.50 g/kg) > α-tocotrienol (465.39 g/kg). The average digestibility of total tocols was 526.78 g/kg.

The contents of micellar, i.e., bioaccessible, α-, γ-, δ-tocopherol, γ-tocotrienol, and total tocols correlated positively with the contents of these compounds in the grain (Table 3).

**Figure 3:**
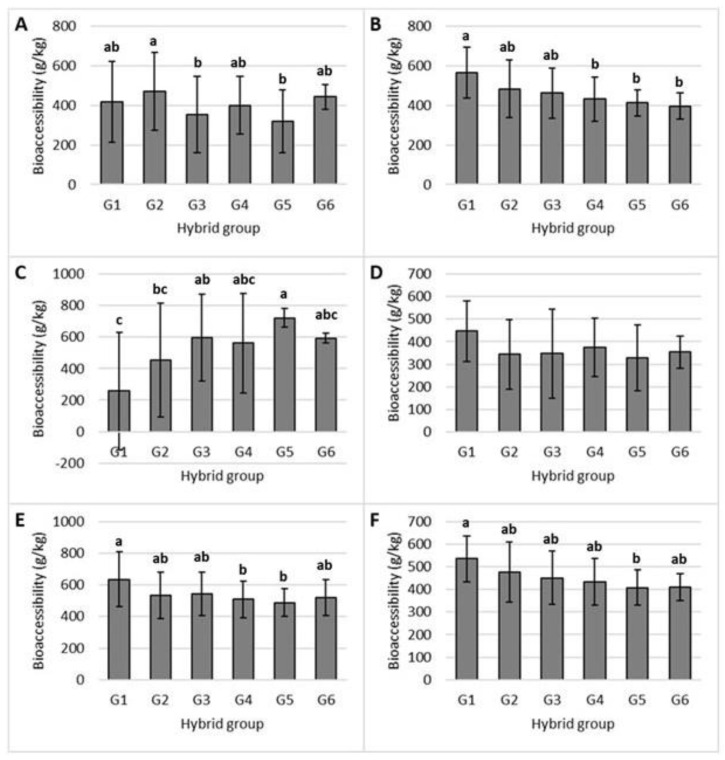
Bioaccessibility of α-tocopherol (**A**), γ-tocopherol (**B**), δ-tocopherol (**C**), α-tocotrienol (**D**), γ-tocotrienol (**E**), and total tocols (**F**) in different maize hybrids, which have been categorized into six groups (G) based on their total vitamin E contents (G1 < 25, G2 25–30, G3 30–35, G4 35–40, G5 40–45, and G6 > 45 μg/g DM). The presented values that are followed by different lower-case letters (a–c) are significantly different from each other (*p* < 0.05).

The range of bioaccessible tocols (µg/g DM) was between 0 and 3.87 for α-tocopherol, between 6.20 and 22.75 for γ-tocopherol, between 0 and 1.08 for δ- tocopherol, between 0 and 0.66 for α-tocotrienol, between 0.41 and 2.12 for γ-tocotrienol, and between 8.59 and 27.72 for total tocols. On average, 454.78 g/kg of the total vitamin E compounds were bioaccessible in the grain of studied commercial maize hybrids. γ-tocotrienol exhibited the highest bioaccessibility (532.77 g/kg), followed by δ-tocopherol (529.88 g/kg), γ-tocopherol (461.76 g/kg), and α-tocopherol (406.49 g/kg). α-tocotrienol exhibited the lowest bioaccessibility, at 359.07 g/kg. Similar to the content in grain, differences were also observed between the hybrid groups, except in the case of α-tocotrienol (Figure 3).

The bioaccessibility of γ-tocopherol and total tocols decreased linearly with the increase in total tocol content in maize grain (*p* < 0.05). This linear effect was confirmed with a negative correlation between grain content and bioaccessibility for both γ-tocopherol and total tocols (Table 4). Negative correlations were also found for α-tocotrienol and γ-tocotrienol, while the bioaccessibility of δ-tocopherol increased linearly with the increase in tocol content in the maize hybrids (*p* < 0.01).

## 3. Discussion

Determination of the tocol profile in maize grain is mainly focused on breeding efforts, while numerous commercial maize hybrids are on the market without information on their tocol content. Therefore, to obtain comprehensive information on tocopherols and tocotrienols, this study analyzed commercially available maize hybrids, which are mainly used as an energy source in animal production in Croatia. In addition, a bioaccessibility analysis should generally be performed to fully evaluate the potential biological activity of maize tocols. However, studies focusing on maize tocols, especially in terms of their bioaccessibility, are still lacking. Therefore, the grain of 103 commercial hybrids from ten breeding companies was analyzed to ascertain the content and bioaccessibility of tocols.

### 3.1. Content of Tocols in Tested Maize Hybrids

The content of tocols differs among maize hybrids due to genetic variations and environmental and other agricultural factors [1]. The commercial hybrids analyzed in the present study had a lower range of tocols than the four inbred lines used in the study by Weber [19] (36.9 to 62.3 µg/g), or in the eight varieties grown at three different locations in Germany for the study by Lux et al. [20] (58.9 to 88.5 µg/g DM), but with a similar range to that of the four genotypes of pigmented maize used in the study by Suriano et al. [21] (16.5 to 42.5 µg/g DM).

Generally, α-tocopherol and γ-tocopherol are the predominant tocol compounds in maize grain, while δ-tocopherol is detected in lower amounts. As for tocotrienols, α- and γ-tocotrienols were found, but they were in much lower amounts than their tocopherol counterparts [15]. In the present study, γ-tocopherol was the predominant vitamin E form found in maize grain, accounting for 75.28% of the total tocols, on average. Other studies reported similar results [16,19,22]. γ-tocopherol has been reported to exhibit several health-promoting effects, such as antioxidant, natriuretic, anti-inflammatory, and chemo-preventive activity [23]. In addition, γ-tocopherol has the ability to scavenge the reactive nitrogen forms [24]. In this study, α-tocopherol was the second most abundant tocopherol, with 12.84% of total tocols being found in this form. Nevertheless, α-tocopherol is reported to be the most active form of vitamin E [7]. In contrast, other forms have lower vitamin E activity: the β-, γ-, and δ-forms of tocopherol have only about 8.1%, 3.4%, and 0.4% of the activity of α-tocopherol, respectively [25]. The genotypes of pigmented maize in the study presented by Suriano et al. [21] differed significantly in tocol profile from the commercial hybrids of yellow maize used in the present study; the most abundant tocol in red maize was α-tocotrienol (75% of total tocols), whereas yellow and purple maize each contained one-third of the total tocols found in α-tocotrienol and γ-tocopherol, respectively. Furthermore, the lower ratio of γ-tocopherol to α-tocopherol is generally expected in genetically modified maize hybrids, as efforts are being made to increase α-tocopherol due to its particularly high bioactivity [26].

As mentioned in Section 2, the contents of γ- and δ-tocopherol and γ-tocotrienol increased linearly with the increase in the content of total tocols (*p* < 0.05) (Figure 1). γ-tocopherol is the predominant form of vitamin E; thus, its linear increase along with the increase in the content of total tocols was expected. A high positive correlation between the contents of ƴ-tocopherols and total tocols in maize was also demonstrated in the study by Goffman and Bohme [16], who reported the highest contribution of this tocol to the total tocol content. Moreover, γ- and δ-tocopherol are formed by the same biosynthetic pathway that proceeds via the common cyclase enzyme, explaining the linearity observed for these two tocols. In contrast, α-tocopherol, which did not show significant linearity with the increase in total tocol content, requires an additional methylation reaction; therefore, it exhibits a different behavior [27]. Likewise, the opposite trends of α- and γ-tocotrienol could also be due to the required methylation of the γ form to obtain α-tocotrienol in the biosynthetic pathway [28].

### 3.2. Bioaccessibility of Tocols in Studied Maize Hybrids

The utilization of tocols in biological functions and their possible deposition in egg yolks depend on their release from the feed matrix. Tocol metabolism in the upper gastrointestinal tract consists of emulsification, micellarization, transport through the mucus layer, and, finally, adsorption by enterocytes [2,29]. Theoretically, the incorporation of tocols into micelles is crucial for its absorption by enterocytes, as it has been demonstrated that the bioaccessibility of lipophilic nutraceuticals generally increases with the increase in the number of mixed micelles [2,18]. Reboul et al. [17] reported extreme variability in terms of vitamin E bioaccessibility, depending on the matrix.

In this study, the standardized INFOGEST procedure was used to determine the bioaccessibility of tocols originating from different maize hybrids. This in vitro digestion method directly reflects the quantity of bioaccessible tocols available for uptake by epithelial cells [30]. After the procedure, the contents of individual and total tocols in the digesta (digestible tocols) and in the micellar fraction (bioaccessible tocols) of the digesta was determined.

The contents of digestible α-, γ-, and δ-tocopherols, γ-tocotrienol, and total tocols increased linearly with the increasing contents of these compounds in the grain. α-tocotrienol was the only tocol that showed no correlation between its content in the grain and the digesta. The content of total digestible tocols was also positively correlated with the content of γ-tocopherol in maize grain. This finding was to be expected since γ-tocopherol is the predominant form of vitamin E in the studied hybrids. Furthermore, tocol compounds with the same biosynthetic pathways demonstrate the same behavior during the digestion phase. High solubilization, i.e., the digestibility of a lipophilic compound, is of great importance since it allows the compound to be further incorporated into micelles and, thus, be bioaccessible [31]. δ-tocopherol exhibited the highest digestibility, while the predominant tocol, ƴ-tocopherol, was ranked third. The average digestibility of total tocols was 526.78 g/kg, which equals approximately 53%. In the study by Mandalari et al. [32], the digestibility of almond tocols averaged 55%, which is consistent with the results obtained for maize in the present study, even though almonds are extremely rich in α-tocopherol rather than ƴ-tocopherol.

The contents of micellar total tocols, α-, γ-, and δ-tocopherols, and γ-tocotrienol increased with the increasing contents of these compounds in the maize grain (Table 3). These positive correlations suggest that the higher the contents of these tocol compounds in the grain, the more of them will be incorporated into the micelles during digestion. In the available literature, only the study by Hossain and Jayadeep [30] evaluated the levels of bioaccessible tocols found in maize. The aforementioned authors found higher levels of bioaccessible tocols in Indian flint-type maize genotypes, in which the γ-tocopherol content (12.57 µg/g DM) was within the range obtained in the present study, while the quantity of bioaccessible tocols decreased in the following order: γ-tocopherol > α-tocopherol > δ-tocopherol > γ-tocotrienol > α-tocotrienol. Among the 103 maize hybrids analyzed in the present study, γ-tocotrienol demonstrated the highest bioaccessibility, while δ-tocopherol demonstrated a similar result (Figure 3). γ-tocopherol had the lowest range of bioaccessibility among the tested hybrids compared to other vitamin E forms. In comparison, Werner and Bohm [33] studied the bioaccessibility of tocols from various types of pasta, and α-tocopherol showed higher bioaccessibility than γ-tocopherol. Therefore, the bioaccessibility of individual tocols varies greatly depending on the matrix used. This was to be expected as large variations in the bioaccessibility of tocols from different matrices have been reported [17]. A similar average result was obtained in the analysis of egg pasta, where the bioaccessibility of total tocols was 49.4% [33]. Hossain and Jayadeep [1] analyzed the tocols found in three maize hybrids; the bioaccessibility of tocopherols and tocotrienols ranged from 42 to 78% and from 50 to 78%, respectively.

Even though an increase in tocol content in maize leads to higher micellarization, which, in turn, results in a higher content of bioaccessible tocols, the results of this study have demonstrated a negative correlation between the bioaccessibility of total tocols, γ-tocopherol, and α- and γ-tocotrienol and the content of these compounds in maize grain (Table 4). The abovementioned inverse relationship suggests that the bioaccessibility of these compounds decreases with increasing content in the grain due to competition for incorporation into micelles, as suggested by Zurak et al. [34] regarding the decrease in the bioaccessibility of carotenoids at higher contents in maize grain.

Numerous factors, such as the lipid phase type and the concentration in the micelles, the surface area and dimensions of micelles, emulsifier type, interfacial properties, and the physical state of micelles, may affect the bioaccessibility of lipid-soluble compounds [35]. Yang and McClements [2] studied the bioaccessibility of α-tocopherol and α-tocopherol acetate in oil-in-water emulsions, using either medium-chain triacylglycerols (MCT) or long-chain triacylglycerols (LCT) as the carrier oil. The LCT system enabled higher bioaccessibility than the MCT emulsion for both α-tocopherol and α-tocopherol acetate. This is probably due to the ability of the long-chain fatty acids in LCT emulsions to accommodate large lipophilic molecules such as vitamin E compounds, as has already been reported in the literature for different lipophilic nutraceuticals [36]. Therefore, the composition of triacylglycerols and fatty acids in the maize hybrids studied should be investigated to better understand their effect on the bioaccessibility of tocols from maize.

## 4. Materials and Methods

### 4.1. Sample Preparation

The present research was conducted on 103 commercial maize hybrids (Table 5). The studied maize hybrids were cultivated in a test field in central Croatia in 2019. Each hybrid was planted on a plot that was 6 m wide and 50 m long. A representative sample weighing 2 kg was taken at harvest for every hybrid. The sample was obtained by combining 5 subsamples that were taken immediately after harvest using a maize harvester. The maize samples were dried at 40 °C to a moisture content below 12%. Afterward, the samples were packed in vacuum-sealed bags and stored at −4 °C. Before analysis, the samples were brought to room temperature, and a portion of the sample was ground in a laboratory mill with a 1 mm sieve (Cyclotec 1093, Foss Tecator, Sweden) for in vitro digestibility, whereas the other fraction was ground in a ball grinder (MM200, Retsch, Haan, Germany) for tocol analysis. The moisture content of the samples was determined by drying at 103 ± 2 °C for 4 h.

### 4.2. In Vitro Digestion

INFOGEST procedure [37] was carried out to evaluate the tocol bioaccessibility in the collected maize hybrids. INFOGEST is a standardized procedure that has already been used to evaluate vitamin E bioaccessibility [38] and the bioaccessibility of other highly lipophilic food micronutrients, such as vitamins D and K and carotenoids [34,38,39,40]. The procedure used in this paper follows that described by Zurak et al. [34]. Even though the aforementioned paper focused on the evaluation of carotenoid bioaccessibility, the procedure could be implemented to determine tocol digestion since it has previously been reported that these two phytochemicals are digested in a highly comparable manner [17]. The method was adapted to mimic the digestion process in the stomach and small intestine of poultry, as described by Weurding et al. [41]; the investigated samples were ground and passed through a 1 mm sieve to mimic the grinding action of the gizzard. Additionally, amyloglucosidase and invertase were used to adapt the procedure to the starch-rich matrix [42].

All the enzymes used in this study were purchased from Sigma-Aldrich (St. Louis, MO, USA). The used enzymes were of porcine origin: α-amylase (A3716, labeled activity ≥ 10 U/mg; experimentally determined at 10 U/mg), pepsin (P7000, labeled activity ≥ 599 U/mg, experimentally determined at 574 U/mg), pancreatin (P7545, labeled activity ≥ 8 USP, experimentally determined trypsin activity at 9 U/mg), invertase (I4504, labeled activity ≥ 300 U/mg), amyloglucosidase (A7095, labeled activity ≥ 260 U/mg), and bile salts (B8631). The content of bile salt added to the reaction mixture containing the enzymes and sample was calculated on the basis that the porcine bile extract contains 50% bile salts, with an average molecular mass of 442 g/mol [43]. 

Oral (simulated salivary fluid, SSF), gastric (simulated gastric fluid, SGF), and intestinal (simulated intestinal fluid, SIF) fluids used in the in vitro digestion procedure were prepared as described by Brodkorb et al. [37]. SSF contained 15.1 mM KCl, 3.7 mM KH_2_PO_4_, 13.6 mM NaHCO_3_, 0.15 mM MgCl_2_(H_2_O)_6_, 0.06 mM (NH_4_)_2_CO_3_, and 1.1 mM HCl. SGF was prepared with 6.9 mM KCl, 0.9 mM KH_2_PO_4_, 25 mM NaHCO_3_, 47.2 mM NaCl, 0.12 mM MgCl_2_(H_2_O)_6_, 0.5 mM (NH_4_)_2_CO_3_, and 15.6 mM HCl. SIF contained 6.8 mM KCl, 0.8 mM KH_2_PO_4_, 85 mM NaHCO_3_, 38.4 mM NaCl, 0.33 mM MgCl_2_(H_2_O)_6_, and 8.4 mM HCl. To simulate the oral phase, a maize sample (1.25 g) was mixed with 1.25 mL of ultrapure water, 2 mL of SSF with a pH of 7, 0.25 mL of α-amylase solution (1500 U/mL in ultrapure water), 12.5 μL of 0.3 M CaCl_2_, and ultrapure water to reach 5 mL. After incubation for 2 min at 37 °C, with horizontal agitation, 4 mL of SGF (pH 3), 3 μL of 0.3 M CaCl_2_, and 0.5 mL of pepsin solution (40,000 U/mL in ultrapure water) were added to the mixture to simulate the gastric phase. The pH was adjusted to 3 with 6 M HCl, and ultrapure water was added to 10 mL. The solution was incubated for 2 h at 37 °C with horizontal agitation. Aiming to mimic the intestinal phase, 4.25 mL of SIF (pH 7), 20 μL of 0.3 M CaCl_2_, and 2.5 mL of an enzyme mixture containing pancreatin (800 U/mL), amyloglucosidase (13 U/mL), and invertase (0.6 U/mL) were added to the mixture. The pH was adjusted to 7 with 1 M NaOH, and the volume was adjusted to 20 mL with ultrapure water. The prepared mixture was incubated for 3 h at 37 °C, with horizontal agitation. After the incubation, the test tubes were placed in an ice bath to stop the intestinal digestion process.

The bioaccessibility of individual and total tocols was calculated according to their content recovered in the micellar fraction after the in vitro digestion procedure, with respect to the content of the tocol in the grain of maize hybrids. The micellar fraction was taken as the fraction obtained after centrifugation [44]. Each hybrid was subjected to the digestion procedure in triplicate on two separate days, and the mean values of bioaccessibility of both the individual and the total tocols were taken as a result.

### 4.3. Tocol Extraction from Whole Maize Grain

Tocols contained in the whole maize grain were extracted as described by Kurilich and Juvik [45]. Firstly, the maize samples (0.6 g) were homogenized with 6 mL of ethanol containing 0.1% of butylhydroxytoluene (BHT) and were incubated for 5 min at 85 °C. Subsequently, the samples were saponified with 100 μL of 80% KOH for 10 min at 85 °C. After the samples were cooled by adding 3 mL of chilled ultrapure water, the liquid-liquid extraction of tocols was performed using 3 mL of *n*-hexane. The phases were separated by centrifugation for 10 min at 2200× *g* (Centric 322A, Tehtnica, Slovenia). The upper layer was separated and the extraction procedure was repeated until a colorless upper layer was obtained (usually after five extraction steps). The collected and combined hexane extract was dried using a rotary vacuum concentrator (RVC 2-25CD Plus, Martin Christ, Germany). Prior to HPLC analysis, the samples were dissolved in 0.2 mL of acetonitrile:dichloromethane:methanol (45:20:35, *v*/*v*/*v*) solution containing 0.1% BHT. Each hybrid was analyzed in triplicate and the mean values of both the individual and total tocols were taken as the result.

### 4.4. Tocol Extraction from the Micellar Fraction

Subsequent to maize digestion, a 5-mL aliquot of the digesta was used directly to extract the digestible tocols. Another aliquot of digesta (8 mL) was centrifuged at 3200× *g* for 1 h at 4 °C and was used for the extraction of bioaccessible tocols, as described by Zurak et al. [34]. In both fractions, tocols were extracted with 2.5 mL of n-hexane in a liquid-liquid extraction procedure, and phase separation was achieved via centrifugation. The upper hexane layer was collected and the extraction was repeated three times. The combined hexane phase was evaporated using a rotary vacuum concentrator. Prior to HPLC analysis, the samples were dissolved in 0.2 mL of acetonitrile:dichloromethane:methanol (45:20:35, *v*/*v*/*v*) solution containing 0.1% BHT.

### 4.5. HPLC Analysis

Tocols in the grain, digesta, and micellar fraction were identified and quantified using a SpectraSystem HPLC instrument (Thermo Separation Products, Inc., Waltham, MA, USA) equipped with a quaternary gradient pump (P4000), an autosampler (AS3000), and an FL detector (FL3000). Tocol separation was achieved with the isocratic elution of the mobile phase on two sequentially connected C18 columns: a Vydac 201TP54 column (5 µm, 4.6 × 150 mm; Hichrom, Reading, UK) and a Zorbax RX-C18 column (5 µm, 4.6 × 150 mm; Agilent Technologies, Santa Clara, CA, USA). The aforementioned columns were protected by a Supelguard Discovery C18 guard column (5 µm, 4 × 20 mm; Supelco, Bellefonte, PA, USA). The mobile phase used was an acetonitrile:dichloromethane:methanol (75:20:5, *v*/*v*/*v*) solution containing 0.1% BHT and 0.05% triethylamine. The flow rate was 1.8 mL/min and 30 µL of the sample was injected. The tocols were monitored at an extinction of 290 nm and an emission of 330 nm.

Tocols were identified by comparing their retention times and were quantified via external standardization with calibration curves, using commercially available standards (Sigma-Aldrich, St. Louis, MO, USA; purity ≥ 96%; r^2^ ≥ 0.98 for all tocols). The total tocol content was calculated by summing the contents of the individual tocols.

### 4.6. Statistical Analysis

The obtained results were analyzed using SAS statistical software (version 9.4; SAS Institute Inc., Cary, NC, USA). Based on the total tocol content in the maize grain, hybrids were classified into the following six groups: G1 (<25 μg/g DM), G2 (25–30 μg/g DM), G3 (30–35 μg/g DM), G4 (35–40 μg/g DM), G5 (40–45 μg/g DM), and G6 (>45 μg/g DM). Differences between the hybrid groups were subjected to an analysis of variance using the MIXED procedure. Means were defined using the least squares means statement and were compared using the PDIFF option; letter groups were determined using the PDMIX macro procedure. Additionally, equally spaced orthogonal contrasts tested the linear, quadratic, and cubic polynomials for a quantitative relationship between the hybrid group and the tocol content in maize hybrids. The contents of tocols in the whole grain, digesta, and micellar fraction and the bioaccessibility of tocols were assessed using a Pearson correlation, as implemented in the CORR procedure. The threshold for statistical significance was defined as *p* < 0.05.

## 5. Conclusions

The studied commercial maize hybrids varied in terms of the contents of individual and total tocols, as was expected due to genotype variability in maize hybrids. The content of total tocols ranged between 19.24 and 54.44 µg/g DM, while the majority of hybrids had a tocol content ranging between 30 and 35 µg/g DM. Accounting for 75% of the total tocols, the predominant form of vitamin E in all the hybrids studied was γ-tocopherol. The standardized INFOGEST procedure allowed the determination of vitamin E content in the digesta and micelles, which is crucial for enabling their adsorption into the epithelial cells of the digestive tract. The contents of the individual and total tocols incorporated into the micelles increased with their increasing contents in the grain of the studied hybrids. Conversely, the bioaccessibility of most tocol compounds decreased with the increasing content in the grain. This resulted in an average bioaccessibility of 454.78 g/kg for total tocols in the studied commercial maize hybrids.

Information on tocol content, let alone on the bioaccessibility of tocols in commercial maize hybrids, is quite sparse. Therefore, the results presented herein could be of great importance for both research purposes and for direct application in animal nutrition. The large variability of the content and bioaccessibility of tocols among maize hybrids allows the selection of hybrids suitable for producing functional eggs. Vitamin E deficiency is still a problem, especially in developing countries. Since eggs are commonly incorporated into the diet of most of the world’s population, the production of vitamin E-enriched eggs could help to reduce vitamin E deficiency and improve the overall health of the population due to the health-promoting benefits of tocols. Furthermore, since maize is already widely used as a livestock feed, factors related to the maize grain that could improve tocol bioaccessibility should be further investigated.

## Figures and Tables

**Table 1:** The proportion of hybrids found in the different groups (G), categorized based on the total tocol content.

Group	Total Tocol Content (µg/g)	Proportion (%)
G1	<25	6.67
G2	25–30	30.48
G3	30–35	25.71
G4	35–40	25.71
G5	40–45	7.62
G6	>45	3.81

**Table 2:** Correlation between the contents of α-tocopherol (αTP), ƴ-tocopherol (ƴTP), δ-tocopherol (δTP), α-tocotrienol (αTT), ƴ-tocotrienol (ƴTT), and total tocols in maize grain and their content in the digesta after the in vitro digestion procedure.

Content of Tocols in Maize Hybrids	Content of Digestible Tocols					
	αTP	γTP	δTP	αTT	γTT	Total Tocols
αTP	0.54 ***	NS	NS	0.23 *	NS	0.26 **
γTP	NS	0.54 ***	0.58 ***	NS	NS	0.48 ***
δTP	NS	NS	0.75 ***	NS	NS	NS
αTT	NS	NS	NS	NS	NS	−0.20 *
γTT	−0.24 *	−0.20 *	0.22 *	NS	0.68 ***	NS
Total tocols	NS	0.52 ***	0.59 ***	NS	NS	0.49 ***

* *p* < 0.05; ** *p* < 0.01; *** *p* < 0.0001; NS—not significant.

**Table 3:** Correlation between the contents of α-tocopherol (αTP), ƴ-tocopherol (ƴTP), δ-tocopherol (δTP), α-tocotrienol (αTT), ƴ-tocotrienol (ƴTT), and total tocols in maize grain and their contents in the micelles after the in vitro digestion procedure.

Content of Tocols in Maize Hybrids	Content of Bioaccessible Tocols					
	αTP	γTP	δTP	αTT	γTT	Total Tocols
αTP	0.52 ***	NS	NS	NS	NS	0.24 *
γTP	NS	0.54 ***	0.50 ***	NS	NS	0.46 ***
δTP	NS	NS	0.71 ***	NS	NS	NS
αTT	NS	−0.27 **	NS	NS	NS	−0.20 *
γTT	−0.21 *	NS	0.20 *	NS	0.72 ***	NS
Total tocols	NS	0.53 ***	0.51 ***	NS	NS	0.48 ***

* *p* < 0.05; ** *p* < 0.01; *** *p* < 0.0001; NS—not significant.

**Table 4:** Correlation between the contents of α-tocopherol (αTP), ƴ-tocopherol (ƴTP), δ-tocopherol (δTP), α-tocotrienol (αTT), ƴ-tocotrienol (ƴTT), and total tocols in maize grain and their bioaccessibility, determined using the in vitro digestion procedure.

Content of Tocols in Maize Hybrids	Bioaccessibility					
	αTP	γTP	δTP	αTT	γTT	Total Tocols
αTP	NS	0.24 *	NS	NS	NS	NS
γTP	NS	−0.32 **	0.28 **	NS	NS	−0.27 **
δTP	NS	−0.35 **	0.27 **	−0.21 *	−0.22 *	−0.30 **
αTT	NS	−0.21 *	NS	−0.47 ***	−0.36 **	−0.24 *
γTT	NS	−0.32 **	NS	−0.29 **	−0.42 ***	−0.31 **
Total tocols	NS	−0.31 **	0.26 **	NS	NS	−0.28 **

* *p* < 0.05; ** *p* < 0.01; *** *p* < 0.0001; NS—not significant.

**Table 5:** List of the studied commercial maize hybrids.

Company	Hybrid	Company	Hybrid	Company	Hybrid
Bc Institut	Agram	LG	LG 30.3115	PIO	Os 3150
Bc Institut	Alibi	LG	LG 30.315	PIO	Os 3450
Bc Institut	Bc 323	LG	LG 31.322	PIO	Os 378
Bc Institut	Bc 344	LG	LG 31.377	PIO	Os 398
Bc Institut	Bc 415	LG	LG 31.545	PIO	Os 4014
Bc Institut	Bc 418	LG	LG 368/08	PIO	Os 4015
Bc Institut	Bc 424	LG	Shannon	PIO	Os 403
Bc Institut	Bc 525	MAS seeds	MAS 34B	PIO	Os 522
Bc Institut	Bc 572	MAS seeds	MAS 48L	PIO	Os 3850
Bc Institut	Instruktor	MAS seeds	MAS 56A	PIO	Posavac 36
Bc Institut	Kekec	NS seme	NS 3022	PIO	Velimir
Bc Institut	Majstor	NS seme	NS 4015	RWA	ES Inventive
Bc Institut	Pajdaš	NS seme	NS 4051	RWA	Ajowan
Bc Institut	Tesla	NS seme	NS 6102	RWA	Inclusiv
Bc Institut	Thriler	NS seme	NS Haris	RWA	Persic
Dekalb	DKC 4670	Pioneer	P0023	RWA	Gladiator
Dekalb	DKC 4920	Pioneer	P0164	RWA	Glumanda
Dekalb	DKC 4943	Pioneer	P0200	RWA	Ulyxxe
Dekalb	DKC 5031	Pioneer	P0216	RWA	Hexagon
Dekalb	DKC 5068	Pioneer	P0217	RWA	Tweetor
Dekalb	DKC 5075	Pioneer	P0412	RWA	Urbanix
Dekalb	DKC 5093	Pioneer	P0725	Syngenta	Sy Andromeda
Dekalb	DKC 5182	Pioneer	P9241	Syngenta	Sy Atomic
Dekalb	DKC 5685	Pioneer	P9300	Syngenta	Sy Bilbao
Dekalb	DKC 5830	Pioneer	P9363	Syngenta	Sy Carioca
KWS	Balasco	Pioneer	P9415	Syngenta	Sy Chorintos
KWS	Kapitolis	Pioneer	P9757	Syngenta	Sy Kreon
KWS	Kollegas	Pioneer	P9889	Syngenta	Sy Lucius
KWS	Kolumbaris	Pioneer	P9903	Syngenta	Sy Photon
KWS	Konfites	Pioneer	P9911	Syngenta	Sy Premeo
KWS	Kashmir	Pioneer	P9978	Syngenta	Sy Sandro
KWS	Orlando	PIO	Tomasov	Syngenta	Sy Senko
KWS	KxB 8386	PIO	Jablan	Syngenta	Sy Zoan
KWS	KxB 8453	PIO	Kulak		
KWS	Smaragd	PIO	Os 3114		

Bc Institut—Bc Institute for breeding and seed production of field crops, Zagreb, Croatia; Dekalb—Group Bayer, Leverkusen, Germany; LG—LG seeds, Westfield, CA, USA; KWS—KWS SAAT SE & Co. KGaA, Einbeck, Germany; MAS seeds—Maïsadour Cooperative Group, Haut-Mauco, France; NS seme—Institute of Field and Vegetable Crops, Novi Sad, Serbia; Pioneer—Corteva Agriscience, Johnston, IA, USA; PIO—Agricultural Institute Osijek—Osijek, Croatia; RWA—Group Bayer, Leverkusen, Germany; Syngenta—Basel, Switzerland.

## Data Availability

The authors confirm that the data supporting the findings of this study are available within the article.

## Supplementary Materials

The following supporting information can be downloaded at: https://www.mdpi.com/article/10.3390/molecules28135015/s1, Table S1: The contents of individual and total tocols in maize hybrids, the digesta, and the bioaccessible fraction for each hybrid.
